# Extraction and Identification of Antibacterial Secondary Metabolites from Marine *Streptomyces* sp. VITBRK2

**Published:** 2014

**Authors:** Benita Mercy Rajan, Krishnan Kannabiran

**Affiliations:** Division of Biomolecules and Genetics, School of Biosciences and Technology, VIT University, India.

**Keywords:** *Streptomyces* sp. VITBRK2, drug resistance, anti-MRSA activity, anti- VRE activity, indoloco-mpounds

## Abstract

Actinomycetes were isolated from marine sediment samples collected from the east coast of Chennai, Tamil Nadu, India. Well diffusion and agar plug methods were used for the evaluation of antibiotic production by these isolates against drug resistant Methicillin- resistant* Staphylococcus aureus* (MRSA) and vancomycin resistant *Enterococci* (VRE). The potential isolate VITBRK2 was mass cultured for morphological and physiological characterization. The culturing conditions of the isolate were optimized and the recommendations of International *Streptomyces *Project were followed for the assimilation of carbon and nitrogen sources. The isolate was identified by comparing the properties with representative species in the key of Nonomura and Bergey’s Manual of Determinative Bacteriology. Ethyl acetate extract prepared from the cell free culture broth of the isolate was analyzed using HPLC- diode array technique to characterize the metabolites and identify the antibiotics. VITBRK2 was found to be Gram-positive rod grey color aerial mycelium production. It was also non motile in nature with spiral spore chain morphology. VITBRK2 was identified as *Streptomyces* and designated as *Streptomyces* sp. VITBRK2. HPLC-DAD analysis showed the presence of indolo compounds (3- methyl-indole and 2-methyl- indole) along with amicoumacin antibiotic. The observed activity of *Streptomyces* sp. VITBRK2 against MRSA and VRE strains may be due to the presence of indolo compounds in the isolate. The results of this study suggested that secondary metabolites produced by *Streptomyces* sp. VITBRK2 could be used as a lead to control drug resistant bacterial pathogens.

Multi-drug-resistant (MDR) bacteria including *Staphylococcus aureus* and *Enterococcus* constitute a serious problem in hospital environments, which require new active antibiotics of wide spectrum (1). Serious infections caused by bacteria have become resistant to commonly used antibiotics and become a major health problem globally in the 21st century (2). Methicillin- resistant* Staphylococcus aureus* (MRSA) is a pathogen responsible for a wide range of infections such as boils, pneumonia, osteomyelitis, endocarditis, bacteremia, etc. and has developed resistance to the majority of conventional antibiotics (3). For more than two decades, these strains were controlled with vancomycin. However, there is an increased incidence of emergence of antibiotic-resistant strains (4, 5). The problem of antibiotic resistance is further complicated by the emergence of other Gram- positive and Gram-negative MDR strains. This multi-drug resistance include amino glycosides, macrolides or fluoroquinolones and the first, second and third generation of penicillin and cephalosporin.

*Enterococcus* infections are caused mostly by vancomycin resistant *Enterococcus* *faecalis*, although in recent years *E. faecium* has also emerged as an important nosocomial pathogen not only resistant to ampicillin/amoxicillin as *E. faecalis* but also much more resistant to vanco-mycin (6). In recent years investigations have been focused to develop active drugs against the ever growing number of resistant pathogens (7). In the year 2001, according to the World Health Organiza-tion (WHO), excessive prescribing and misuse of antibiotics has led to the resistance of many pathogens (8). Today, new drug-resistant strains appear more quickly, while the rate of discovery of new antibiotics has decreased significantly.

In recent years new therapeutic agents have entered in the clinical area, unfortunately with some side effects (9-10). Side effects of existing drugs and drug resistance have become serious public health problems which require the development of new antimicrobial agents (11-12). Currently, many scientists are working on new antimicrobial drugs, mainly of actinomicetal origin (13). Actinomycetes are producers of metabolites with antimicrobial, anti-parasite, antiviral, antitumor activity, cytotoxic, etc; whose chemical structures are unique (14). Until recently marine sediments as a source of bioactive actinomycetes have remained as the least explored resources, but today it becomes one of the more promising sources. *Streptomyces* are a prolific source of secondary metabolites yielded many antibiotics; more than 80% of antibiotics available in the market are from *Streptomyces* (15). In the present study, we report the antibacterial activity of *Streptomyces* isolated from marine sediments against MRSA and VRE strains.

## Materials and Methods

**Sample collection and isolation of actinomycetes**

Marine sediment samples were collected from east coasts of Chennai, Bay of Bengal, India at a depth of 400 cm. The sediment samples were dried in laminar air flow for 8-12 h and then kept at 42°C for 10-30 days in a sterile Petri dish and these preheated samples were used for the isolation of actinomycetes. The International Streptomyces Project (ISP) No. I media, Starch casein agar and Bennett’s agar with 25% sea water, 25% sediment extract was used for the isolation of actinomycetes and the growth media was supplemented with antibiotics, cycloheximide (25 mg/ml) and nalidixic acid (25 mg/ml) (Himedia, Mumbai, India). Plates were incubated at 28°C for 7-14 days. The isolates were subcultured and maintained in slant culture at 4°C as well as in 20 % (v/v) glycerol stock at -80 °C.

**Bacterial strains**

Gram-positive bacterial MRSA strains *Staphylococcus aureus* (ATCC 29213), *Staphyl-ococcus aureus *(ATCC 25923), *Staphylococcus aureus *(ATCC 700699) and *Staphylococcus aureus *(U2A 2150) were chosen for this study. VRE bacterial strains, *Enterococcus faecalis* (ATCC 29212) and *Enterococcus faecium *(BM4107) were obtained from ATCC culture collection center. The drug resistant strains *Enterococcus faecium *(BM4147-*Van A*) were obtained from Pasteur Institute; Paris.

**Screening for antibiotic production**

Antibacterial activity of the potential isolate was studied by agar plate diffusion assay. Briefly, 10µl of the cell free supernatant was applied to filter disks (6mm in diameter) (16). Inhibition zones were expressed as diameters and measured after incubation at 37°C for 24h. All actinomyces isolates were screened for antibacterial activity against all seven ATCC drug resistant strains. Influence of the various culture media on the antibacterial potential of the isolate was studied by cylinder plug method using ISP 1 supplemented with seawater collected at the sampling site, marine agar, actinomycetes isolation agar, starch casein agar (Himedia, Mumbai, India).

**Characterization and identification of the pote-ntial isolate**

The morphological, cultural, physiological and biochemical characterization of the potential isolate was carried out as described in ISP (17). The morphology of the spore bearing hyphae with the entire spore chain with the substrate and aerial mycelium of the strain was examined by light microscope (1000x magnification) as well as scanning electron microscope (Hitachi, S-3400N). Media used were those recommended in the ISP (18). Mycelium was observed after incubation at 28°C for 2 weeks and colors were also determined. Carbohydrate utilization was determined by growth on carbon utilization medium (ISP 9) (19) supplemented with 1% carbon sources at 28°C. Temperature range for growth was determined on inorganic salts starch agar medium (ISP 4) using a temperature gradient incubator. Hydrolysis of starch and milk were evaluated by using the glucose starch agar and skim milk agar, respectively. Reduction of nitrate and production of melanin pigment were determined by the ISP method (20). All cultural characteristics were recorded after 14 days.

**Optimization of nutritional and cultural conditions**

In order to optimize the nutritional and cultural conditions and to identify the suitable media for growth, the strain was inoculated in different culture media (SCA, ISP 2, ISP 3, ISP 4, ISP 5, ISP 6, ISP 7, modified Bennett’s agar, sucrose/ nitrate agar, and nutrient agar) and the growth was investigated. The effect of cultural conditions like different incubation temperatures (15, 25, 37 and 50°C), different pH (5.0, 6.0, 7.4 and 9.0) and NaCl concentrations (2, 5, 7, 9 and 12%) on the growth of the isolate was also studied. The carbon and nitrogen sources required were also studied by inoculating the isolates into mineral salt agar with different sugars substituted to starch (D-glucose, sucrose, starch, D-xylose, D-galactose, maltose, L-arabinose, fructose, lactose, and glycerol), organic nitrogen sources like peptone, yeast extract, casein and inorganic sources like ammonium sulphate, ammonium nitrate and urea. The concentrations of carbon sources and carbon utilization tests were done as described earlier (21, 22). After incubation, the dry weight of the mycelium was measured and correlated with the growth of the isolate. Based on the growth of the isolate in different media, the cultural conditions were optimized. 

**Fermentation and extraction of secondary met-abolites**

Spores (10^7^/ml) of the isolate were used to inoculate 1000 ml Erlenmeyer flasks containing 200 ml of ISP 1 broth supplemented with 1% (w/v) of glucose and magnesium. After incubation at 30°C for 24 h in an orbital incubator shaker at 200 rpm, this pre-culture was used to inoculate (5% v/v) 15 L culture medium having the same composition as the pre-culture. After six days of incubation, the culture broth was filtered to separate mycelium and supernatant, the mycelium was lyophilized, extracted with acetone and concentrated on a rotary evaporator. The supernatant was extracted twice with equal volume of ethyl acetate and the combined organic layers were evaporated to obtain the ethyl acetate extract (EA extract).

**HPLC-DAD analysis of the EA extract**

A total volume of 15 litters of the culture broth was centrifuged in batches for 15 min at 10000 rpm and the cell free supernatant was extracted with equal volume of ethyl acetate. The solvent fraction was collected and evaporated to dryness in vacuum and re-suspended in 1 ml of ethanol. The solvent was allowed to evaporate and the residue was lyophilized. The obtained EA extract was subjected to HPLC-DAD screening (University of Tubingen, Germany). The HPLC-DAD chromatographic system consisted of an HP 1090M liquid chromatography equipped with a diode-array detector and HP Kayak XM 600 Chem Station (Agilent Technologies). Multiple wavelength monitoring was performed at 210, 230, 260, 280, 310, 360, 435 and 500 nm. The UV-visible spectrum was measured from 200 to 600 nm. Sample (5 μl) was injected onto an HPLC column (125 X 4.6 mm, guard column 20 4.6 mm) filled with Nucleosil-100 C-18 (5 m). Separation was performed by a linear gradient using 0.1% orthophosphoric acid as solvent A and acetonitrile as solvent B. The gradient was from 0 to 100% solvent B in 15 min at a flow rate of 2 ml/min. Limitations of the method are as follows: polar compounds cannot be separated because of non-retention behaviour on the reversed-phase column. These compounds show front elution. Only the compounds having a UV active chromophore can be detected. Sugar type compounds (e.g. aminoglycosides) or peptides containing aliphatic amino acids cannot be detected by this method.

## Results

A total of 120 actinomycetes were isolated from marine sediment samples collected at different locations of Bay of Bengal cost if Chennai, Tamil Nadu, India. Out of 120 isolates, the isolate named as VITBRK2 was found to have significant antibacterial activity against standard MRSA and VRE strains. The potential isolate VITBRK2 showed antagonistic activity against MRAS and VRE strains were chosen for further studies.

The culture supernatant (filtrate) of the isolate showed significant antibacterial activity against MRSA strains with the zone of inhibition of 17 mm against *S. aureus* (ATCC 29213), 21 mm against *S. aureus *(ATCC 25923), 15 mm against *S. aureus *(ATCC 700699) and 12 mm against *S. aureus* (U2A 2150) (Table.1). It also showed antibacterial activity against VRE strains, 23 mm zone of inhibition against *E. faecalis* (ATCC 29212), 19 mm against *E. faecium *(BM4107) and 21 mm against *Enterococcus faecium *(BM4147) (Table. 1). All values are the mean of three independent experiments.

**Fig. 1:**
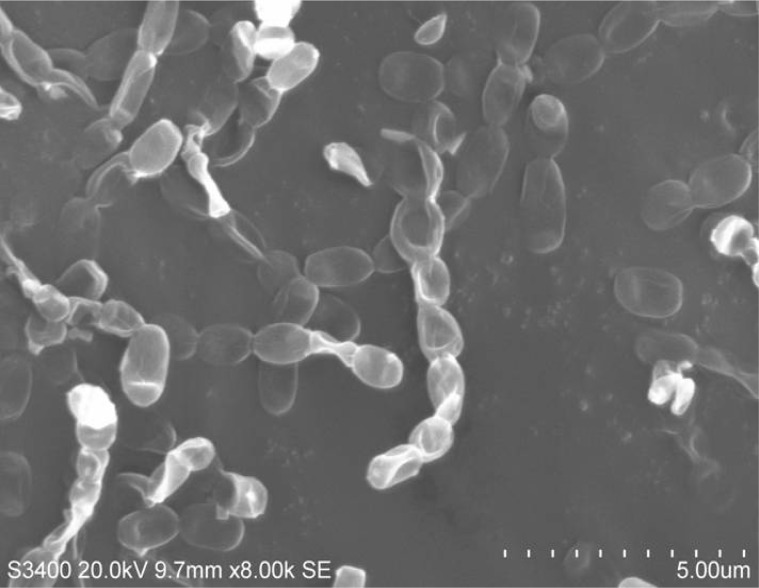
Smooth spore surface morphology of *Streptomyces* sp*.* VITBRK2 observed under scanning electron microscope. The bar represents 5µm.

**Table 1:** Antagonistic activity of VITBRK2 against drug resistant MRSA and VRE strains

**Microbial strains**	**Zone of inhibition (mm)**
*Staphylococcus aureus* ATCC 29213	17
*Staphylococcus aureus* ATCC 25923	21
*Staphylococcus aureus *(700699)	15
*Staphylococcus aureus* U2A 2150- MRSA	12
*Enterococcus faecalis* ATCC 29212	23
*Enterococcus faecium* BM4147-Van A	21
*Enterococcus faecium* BM4107	19

It was observed that the mature sporulating aerial mycelium was whitish grey in color. The spore chain morphology was observed under optical microscope at 1000X magnification. The smooth spore surface morphology was observed under scanning electron microscopic (SEM) analysis (Fig.1). The growth of the isolate was maximal on ISP1 medium supplemented with sea water and its growth was equally maximal on actinomycetes isolation agar as well. The isolate showed maximum growth when cultivated at tempera-ture 28°C; pH 7.4, with seawater 25%. The isolate assimilated arabinose, xylose, inositol, mannitol, fructose, sucrose and raffinose, however, the isolate did not utilize rhamnose (Table.2).

**Table 2:** Cultural characteristics of the isolate *Streptomyces* sp. VITBRK2

**VITBRK2**	**Growth characteristics**
Growth under anaerobic conditions	Negative
Gram stain	Positive
Shape and growth	Long filamentous
Motility	Non-motile
Temperature range for growth	25-37°C
Optimum temperature	28°C
Range of pH for growth	6-8
Growth on MacConky plates	Negative
Growth in the presence of NaCl	2-12 %
2%	Positive
5%	Positive
7%	Positive
9%	Positive
12%	Positive
Aerial mass colour	Whitish grey
Melanoid pigments	Negative
Reverse side pigments	Negative
Soluble pigments	Negative
Spore chain	Spiral
Spore surface	Smooth
Arabinose	Positive
Xylose	Positive
Inositol	Positive
Mannitol	Positive
Fructose	Positive
Rhamnose	Negative
Sucrose	Positive
Raffinose	Positive


**Fig. 2:**
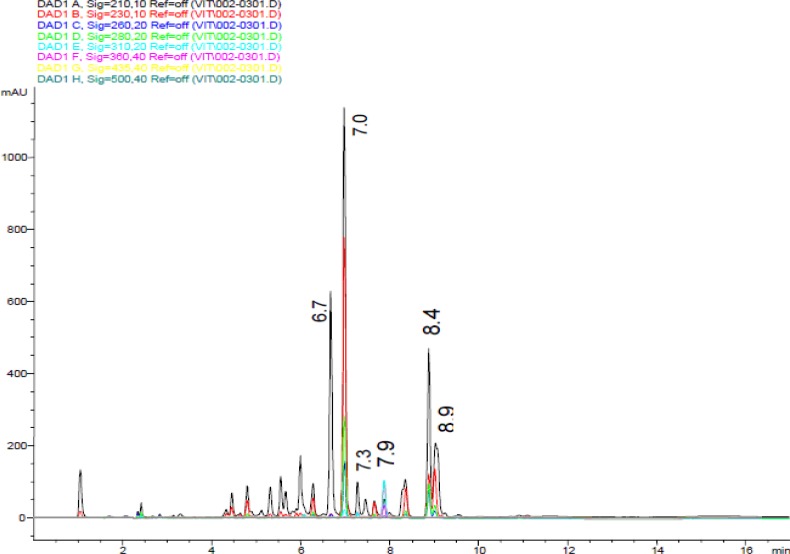
The HPLC-DAD chromatogram of ethyl acetate extract of *Streptomyces* sp. VITBRK2

**Fig. 3:**
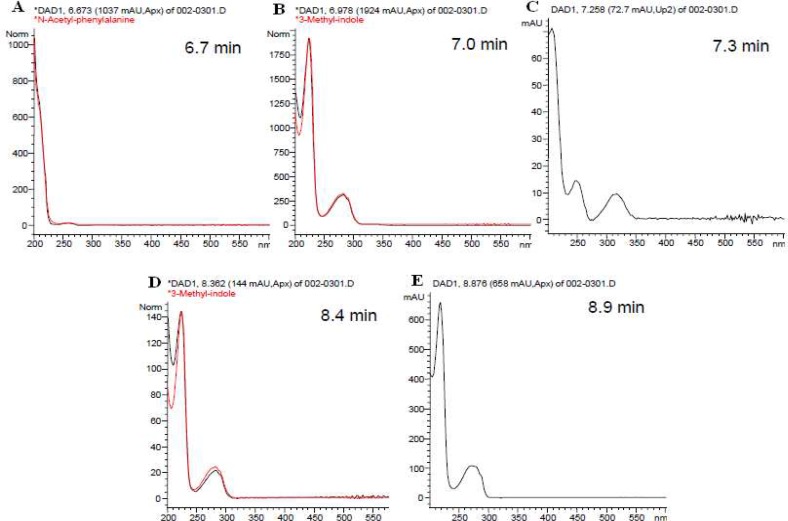
The HPLC-DAD chromatogram of ethyl acetate extract of *Streptomyces* sp. VITBRK2 A) Peak at 6.7 min represents N-Acetyl-phenylalanine B) Peak at 7.0 min corresponds to 3-methyl-indole, C) Peak at 7.3 min represents amicoumacin antibiotic D) Peak at 8.4 min represents 3-methyl-indole and E) Peak at 8.9 represents indole type compound

The isolate utilized 0.1% of L-asparagine, L-phenylalanine, L-histidine and L-hydroxyprolone as nitrogen source. The isolate was halophylic in nature tolerated NaCl concentrations between 2% to 12%. Based on the results of physiological, biochemical and cultural characterization, the isolate was identified as *Streptomyces *and designated as *Streptomyces *sp.VITBRK2.

HPLC-DAD analysis of the EA extract of *Streptomyces *sp. VITBRK2 is shown in Fig.2. The peaks of the chromatogram were matched with the reference compound available in the database by UV-Visible spectrum. The peaks in the chromatogram having the same UV-Visible spectrum and retention time with that of the reference compound was identified and named. In the UV-Visible spectra various peaks observed corresponded to different compounds (Fig.3). Peak at 6.7 min (A) represents N-Acetyl-phenylalanine, 7.0 min (B) 3-methyl-indole, 7.3 min (C) amicoumacin antibiotic, 8.4 min (D) 3-methyl-indole and Peak at 8.9 (E) corresponds to indole type compound.

## Discussion

Antibiotic resistance is reaching a critical level because only few options are available to treat certain pathogenic bacteria mainly those causing hospital-acquired and community acquired infections (23). The potential isolate *Streptomyces *sp.VITBRK2 isolated from marine sediment samples collected at the east coast of Chennai, India exhibited antagonistic activity against drug resistant MRSA and VRE strains. The culture supernatant of the isolate showed significant antibacterial activity against drug resistant standard MRSA and VRE strains. Fractionation of EA extract by HPLC DAD resulted in the identification of in dole type of compounds along with amicoumacin antibiotic. Indolo compounds have shown significant antibacterial activity against drug resistant Gram-positive and Gram-negative bacteria pathogens(23).

*S. aureus* is the etiological agent for a large number of human infections, including pneumonia, meningitis, toxic shock syndrome, bacteremia, and endocarditis. *S. aureus* is notorious for developing rapid resistance to antibiotics, which is caused mainly by antibiotic selection and horizontal transfer of resistance genes (24). Most notably, MRSA emerged quickly due to the acquisition of the novel penicillin- binding protein 2A (PBP2A) encoded by *mecA* (25). New alkaloid, 3-((6-methylpyrazin-2-yl) methyl)-1H-indole isolated from the deep-sea actinomycete *Serinicoccus profundi* sp. nov. had been shown to possess antibactarial activity against *S. aureus* (26). A compound, 2-(2’, 4’-Dibromophenoxy)-4, 6-dibromophenol isolated from the marine sponge *Dysidea granulosa *(Bergquist) collected at the coast of Lakshadweep islands, Indian Ocean, exhibited potent and broad spectrum *in-vitro *antibacterial activity, especially against MRSA, methicillin sensitive *Staphylococcus aureus *(MSSA), VRE, vancomycin sensitive *Enterococci *(VSE) and *Bacillus *spp. (27). Bis-indole compounds, MBX 1066 and 1162 exhibited potent activities against all Gram-positive species which included MRSA and VRE strains (23). Amicoumacin A reported to possess strong antimicrobial activity against MRSA (28). Amicoumacins extracted from marine derived *Bacillus subtilis* strain B1779 was shown to possess strong bactericidal activity against MRSA and VRE strains (29). The results of this study revealed that *Streptomyces *sp. VITBRK2 is the potential actinomycetes isolate capable of producing indolo antibacterial compounds acting against drug resistant MRSA and VRE strains.
